# Left Atrial Function Is Improved in Short-Term Follow-Up after Catheter Ablation of Outflow Tract Premature Ventricular Complexes

**DOI:** 10.3390/medicina55060241

**Published:** 2019-06-03

**Authors:** Selçuk Kanat, Ferit Onur Mutluer, Ahmet Tütüncü, Bilge Duran Karaduman, Veciha Ozlem Bozkaya, Muhammed Keskin, Abdulkadir Uslu, Serkan Çay, Erhan Tenekecioglu

**Affiliations:** 1 Department of Cardiology, Bursa Education and Research Hospital, Health Sciences University Bursa, 16310 Bursa, Turkey; drselcukkanat@gmail.com (S.K.); tutuncuahm@yahoo.com (A.T.); 2 Department of Cardiology, Erasmus MC, Erasmus University, 3000 CA Rotterdam, The Netherlands; onurmd@gmail.com; 3 Department of Cardiology, Atatürk Education and Research Hospital, Yildirim Bayezit University, 06760 Ankara, Turkey; bilge_dr@yahoo.com; 4 Department of Cardiology, Zekai Tahir Burak Education and Research Hospital, 06230 Ankara, Turkey; vobozkaya@gmail.com; 5 Department of Cardiology, Istanbul Sultan Abdulhamid Han Education and Research Hospital, 34668 Istanbul, Turkey; drmuhammedkeskin@gmail.com; 6 Department of Cardiology, Kosuyolu Education and Research Hospital, 34865 Istanbul, Turkey; dr.akadiruslu@gmail.com; 7 Department of Cardiology, Ankara Yüksek İhtisas Education and Research Hospital, 06100 Ankara, Turkey; cayserkan@yahoo.com

**Keywords:** premature ventricular complex, outflow tract, radiofrequency ablation, left atrial function, echocardiography

## Abstract

*Background:* Association of premature ventricular complexes (PVC) with left ventricular systolic dysfunction (LVSD) and efficacy of catheter ablation treatment have been demonstrated in studies. The role of left atrial (LA) mechanics in the etiopathogenesis of PVC-induced cardiomyopathy (PVC-CMP) as well as changes in LA mechanics with catheter ablation have not been studied before. *Methods:* A total number of 61 patients (Mean Age 43 ± 3) with idiopathic outflow tract (OT) PVCs undergoing radiofrequency catheter ablation (RFCA) were enrolled. ECG, 24 h Holter, and echocardiographic evaluation with left ventricular (LV) diastolic functions and LA volumetric assessments were performed before and three months after RFCA. *Results:* Along with a marginal increase in left ventricle ejection fraction (LVEF), improvement in diastolic functions and left atrial mechanics were observed in the study (LVEF 53 ± 7 versus 57 ± 6, *p* < 0.01) in short-term follow-up. The frequency of LV diastolic dysfunction (LVDD) decreased with catheter ablation (*n* = 5 to 0, *p* = 0.02). The overall LA function improved. Left atrium passive and overall emptying fraction (LAEF) increased significantly (0.32 ± 0.04 to 0.41 ± 0.04, *p* < 0.05 and 0.62 ± 0.04 to 0.65 ± 0.004, *p* < 0.05, respectively). Active LAEF decreased significantly (0.29 ± 0.005 to 0.24 ± 0.006, *p* < 0.05). *Conclusions:* The results of this study are indicative of “PVC-induced atriomyopathy” which responds to RFCA in short-term follow-up. Atrial dysfunction might play a role in symptoms and etiopathogenesis of LVSD.

## 1. Introduction

Premature ventricular complexes (PVCs) are not uncommon in the general population and are often an incidental finding in ECG or Holter recordings of healthy individuals. Their clinical significance and consequences depend on various properties of the arrhythmia. Left ventricle systolic dysfunction, which is termed PVC-induced cardiomyopathy (PVC-CMP), is an undesirable consequence associated with frequent PVCs [1].

Most of the patients with PVCs do not need a specific treatment. The presence of intractable symptoms clearly attributable to PVCs, underlying heart disease that would render the patient susceptible to complications of this arrhythmia, a very high PVC burden in 24 h (i.e., >15% of total beats in Holter monitorization) that would increase PVC-CMP risk significantly, or the presence of PVC-CMP at the time of diagnosis warrant a treatment with antiarrhythmics, pacing, or ablation, depending on the underlying etiopathogenesis [2].

Radiofrequency catheter ablation (RFC) has become popular because of its potential of eliminate arrhythmias completely, which would consequently enable a physician to discontinue antiarrhythmics which are associated with several side effects and complications. Additionally, the procedure was associated with improved outcomes when performed for PVC-CMP [3]. Guidelines by the European Heart Rhythm Association (EHRA) recommends that RFCA should be reserved for high PVC burden, intractable symptoms, or PVC-CMP [4]. Premature ventricular complex-induced cardiomyopathy is defined as left-ventricle dysfunction with a left ventricle ejection fraction (LVEF) below 50%. It is a well-defined morbidity associated with PVCs [5].

In Turkey, the major cause of death in the very elderly is cardiovascular disease. In 2016, the rate of death from circulatory system diseases was 46.8% in Turkey, higher than the rate in Europe, which was 42% [6]. Smoking prevalence was 48% in men and 15% in women; hypertension prevalence was approximately 30% in both sexes; obesity prevalence was 13% in men and 33% in women [6].

Abnormalities in left atrium (LA) geometry and volumes as well as the response of these parameters to catheter ablation were studied before in a heterogeneous set of patients with PVC-CMP and demonstrated significant positive changes in diastolic functions and LA volumes [7]. Left atrial mechanics or their response to ablative therapies in patients with idiopathic PVCs were not studied before. In this study, the aim was to unravel the role of outflow tract PVCs (OT-PVCs) and RFCA for this arrhythmia in left atrial functions and LV diastolic functions, by direct assessment of dynamic changes in atrial volumes during the cardiac cycle.

## 2. Methods

### 2.1. Study Population

Sixty-one patients older than 18 years with OT-PVCs in their ECG or 24 h Holter recordings, without significant structural heart disease, who consequently underwent catheter ablation, were enrolled prospectively. Outflow tract PVC was defined as the absence of structural heart disease with a left-bundle branch block, inferior axis, relatively narrow QRS duration, and R/S transformation later than V3 in surface ECG. Patients with ischemic heart disease, more than mild-degree valvular lesion, prosthetic valve, previous history of cardiac surgery, known diagnosis of cardiomyopathy, pericarditis, myocarditis, or endocarditis and patients with non-sustained or sustained ventricular tachycardias or other types of supraventricular tachycardias in rhythm Holter recordings were excluded from the study.

The study was approved by the Ethical Committee of Bursa Education and Research Hospital. Written informed consent was obtained from all patients prior to participation in the study, and this study was conducted in accordance with the Declaration of Helsinki.

### 2.2. Diagnostic Work-Up

All patients underwent a detailed diagnostic work-up including ECG, 24 h Holter monitorization, echocardiography, radionuclide scans, coronary computerized tomographic angiography, and/or invasive coronary angiography and cardiac magnetic resonance imaging as indicated, to rule out coronary artery disease (CAD) and valvular or structural heart disease at least three months prior to the ablation procedure [7].

### 2.3. Echocardiography

Two-dimensional transthoracic echocardiography (Philips i33, Eindhoven, The Netherlands) was performed at least three months prior to the procedure and three months following the ablation procedure. A standard examination protocol was followed by a detailed assessment of the left ventricle (LV) diastolic functions and LA functions [8]. Left ventricle ejection fraction was determined by the biplane Simpson method. Premature ventricular complex-induced cardiomyopathy was defined as left-ventricle dysfunction with an LVEF below 50%. It is a well-defined morbidity associated with PVCs [5].

### 2.4. Determination of Diastolic Functions

Left ventricular diastolic functions (LVDD) were assessed by pulsed-wave Doppler analysis of the diastolic mitral inflow and Tissue Doppler imaging of the left ventricular wall at the basal segments of the lateral and septal walls. Conventional Doppler parameters along with LVDD grading according to the latest guidelines were assessed, calculated, and recorded [8,9].

### 2.5. Left Atrium Volumetric Measurements

Volumetric measurements were performed with a modified Simpson’s method, as defined in the guidelines [10]. Left atrial volumes were recorded as were gated with surface ECG. End-systolic, end-diastolic, and volumes prior to atrial systole were measured with the biplane area-length method. Atrial reservoir, conduit, and booster pump functions were evaluated. Left atrium conduit and booster pump functions were evaluated according to a methodology defined in the literature [11,12]. Volumes were indexed to body surface area estimated from height and weight using the Dubois formula [13]. Volumetric measures other than left atrium volume index (LAVI) were also indexed to body surface area (BSA) when necessary to standardize the results. All the measurements were done at least five beats after a PVC (Table 1).

### 2.6. Follow-Up

Electrocardiography, 24 h Holter recording and 2-dimensional transthoracic echocardiography (2D-TTE) were repeated at three-month follow-up. At least 80% decrease in PVCs with the same morphology in 24 h Holter recording at three months was defined as successful sustained ablation. Antiarrhythmic medications were not started again after the ablation procedure.

### 2.7. Electrophysiology Study and Catheter Ablation

Patients were instructed to discontinue antiarrhythmic drugs for a period of five half-lives prior to the planned ablation procedures. The electrophysiological study was performed during a fasting state, by local anesthesia. The patients were not sedated prior to procedure to avoid the risk of suppressing the automaticity. If clinical premature ventricular complexes (PVCs) were not present at baseline, isoproterenol infusion and electrical stimulation were used to induce the arrhythmia. Intravenous isoproterenol 1–5 μg/min was infused to achieve at least 20% heart rate increment. Electrical stimulation was performed using incremental pacing and triple extra-stimuli pacing from the right ventricular apex or right ventricular outflow tract (RVOT).

Electroanatomical mapping was performed with the EnsiteTM Precision (Abbott, Chicago, IL, USA) or CARTO (Biosense Webster, Irvine, CA, USA). Using fluoroscopic guidance, a decapolar mapping catheter was inserted through the right femoral vein and positioned in the coronary sinus. The ablation catheter used for mapping and ablation was an 8F quadripolar irrigated ablation catheter Thermocool Smarttouch CF (Biosense Webster Inc., Diamond Bar, CA, USA) and FlexAbility (Endosense/Abbott, St Paul, MN, USA) with a 4 mm tip and a 2–5–2 mm inter-electrode spacing. Left-sided access was performed via retrograde aortic route or trans-septal puncture, and intravenous unfractionated heparin was administered to maintain an activated clotting time between 300 and 350 s.

In the first step, an activation map was implemented using a mapping catheter. The PVC focus was determined as the earliest local timing and a QS-complex on unipolar electrogram in case of radial spread away from the focus. The goal of activation mapping was to find the earliest sites ≳30 ms ahead of reference surface QRS onset. Additionally, to identify the origin and optimal ablation site of PVCs, pace mapping was implemented, aiming for at least 11 of 12 leads matching with clinical PVCs. In the outflow tract ventricular zone, radiofrequency energy was delivered in a power-controlled mode at 30–35 W for the irrigated catheter, targeting for an impedance decrease of 10 Ω. Electrophysiologic study and programmed electrical stimulation (PES) were repeated with and without isoproterenol following 30 min of waiting period after ablation, and non-inducibility of tachycardia or (Ventricular premature complexes) VPCs with the same morphology was assumed as successful ablation.

### 2.8. Statistical Analysis

Statistical analysis was performed with SPSS version 22. Continuous variables were summarized as mean ± standard deviation or median (minimum, maximum) when normal distribution was not present. Analysis of variance for repeated measures was used for evaluating changes in echocardiographic parameters after ablation. Wilcoxon signed-rank test was used for comparing the changes in distribution of ordinal values after RFCA. *p*-values of less than 0.05 were accepted to represent statistically significant differences. Analysis of variance for repeated measures procedure was used for assessing the significance of changes in parameters with RFCA along with the effects of various factors on changes.

## 3. Results

A total number of 61 patients with a median age of 41 years (mean age: 43 ± 3, 32/61, 52.5% male) were enrolled in the study. None of the patients had a syncopal episode or documented ventricular tachycardia (VT) in their recent or past medical history. The burden of PVC localized electrocardiographically to outflow tract (OT) was 20% (9–33%) in 24 h Holter recordings. There were no patients with severe left ventricular systolic dysfunction, defined as a LVEF <30% [14]. The characteristics of the study population are shown in Table 2.

The procedural characteristics of the study population are shown in Table 3. Preprocedural complications were tamponade in a patient, managed with pericardiocentesis, cerebrovascular accident in one patient, which resolved with complete recovery, and entry-site hematoma in three patients. None of the hematoma cases needed blood transfusion or further intervention. Premature ventricular complexes were localized to the RVOT in the majority of cases (29/61, 47%).

The effect of RFCA on OT-PVCs was examined in three domains: effects on left ventricle systolic functions, effects on LVDD, and effects on left atrial mechanics, with VPC burden as a covariate, and antiarrhythmic use and prior history of RFCA as cofactors.

Mitral E-wave, A-wave, deceleration time of the E wave (DT), e’-wave, all changed significantly after ablation in directions indicative of improvement in LVDD, whereas E/A ratio did not change significantly, and E/e’ ratio improved only among patients with PVC-CMP at baseline (Table 4). There was a marginal but significant improvement in LVEF in follow-up echocardiography, independent of VES frequency, antiarrhythmic use at initial assessment, and prior history of RFCA (*p* < 0.05) (Table 5). Only a small portion of patients had LVDD, all Grade 1 (5/61, 7.9%). However, none of the patients demonstrated LVDD at follow-up (*p* < 0.05). 

**Figure 1:**
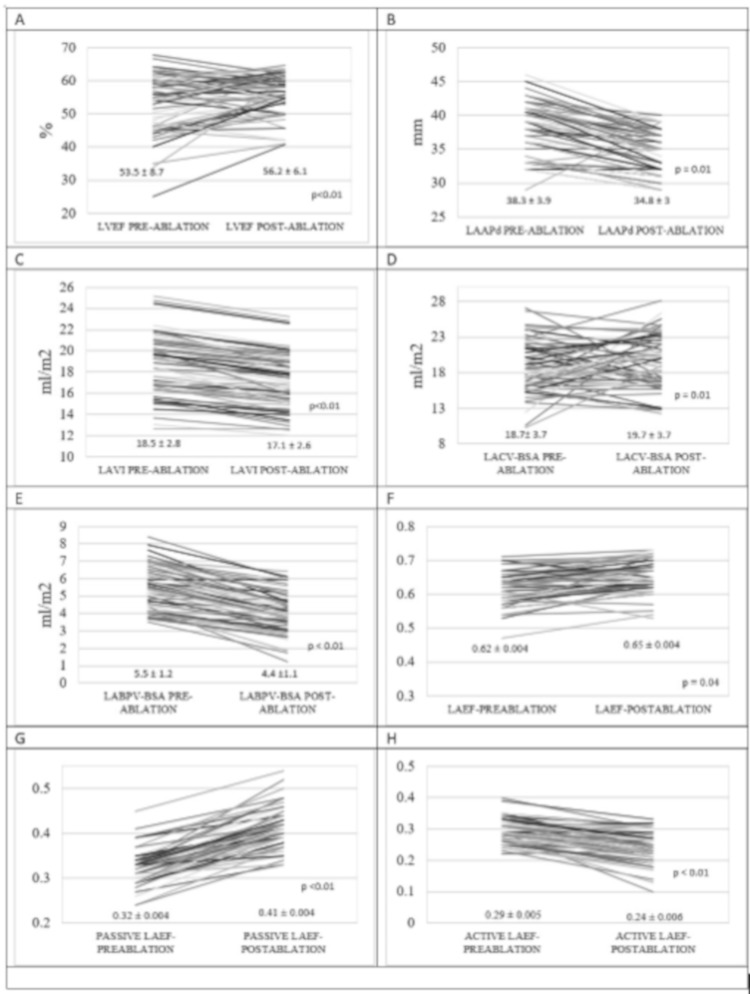
Changes in several selected measures with RFCA. LAAPd: left atrium anteroposterior diameter, LACV: left atrium conduit volume.

Left atrial mechanics revealed statistically significant responses to RFCA along with LV diastolic functions. Left atrium anteroposterior diameter (LAAPd) and LAVI decreased significantly, and left atrium conduit volume indexed to BSA (LACV-BSA) increased significantly after RF ablation compared to pre-ablation values (Table 5). However, left atrium booster pump volume indexed to BSA (LABPV-BSA) paradoxically decreased, and left atrium ejection fraction (LAEF) increased minimally (Figure 1 and Table 4). None of the four factors consisting of PVC burden, antiarrhythmic use, prior ablation history, or baseline LAVI affected changes in left atrial mechanics significantly in multivariate analysis (Table 5).

Subgroup analyses for LVEF and LA mechanical functions were performed in patients with PVC-CMP. There was significant improvement in LV systolic function (Table 6). Left atrium anteroposterior diameter and LAVI decreased significantly after RFCA. LABPV-BSA decreased, and LAEF and passive LAEF increased significantly. However, change in conduit volume was not statistically significant (Table 6).

## 4. Discussion

The main findings from the current study could be summarized as follows: Firstly, left ventricular function improved significantly during short-term follow-up of the patients following RF ablation. Secondly, the frequency of LVDD significantly decreased as a result of RF ablation. Thirdly, parallel to the improvement in left ventricle diastolic functions, significant improvement was observed in left atrial active systolic function, as assessed by left atrial volumes.

The development of tachycardia-induced left ventricular systolic dysfunction (LVSD), independent of accompanying structural heart disease is a well-defined clinical condition [15]. Left ventricular systolic function decreases in a predictable manner in response to tachyarrhythmia. Previous studies have shown a clear correlation between risk and severity of tachycardia-induced LVSD and arrhythmia burden, as well as a significant improvement in LV functions with RF ablation or antiarrhythmic therapy. Few risk factors were defined previously, i.e., high PVC burden (higher than 16%), wide QRS PVCs (>150 ms), epicardial origin, higher coupling interval (115 ± 25 versus 94 ± 19 ms), which all predicted PVC-CMP [4,16,17,18,19].

The efficacy of RFCA in the management of LV systolic function was questioned in several trials, and a favorable effect of this modality on LV as well as RV systolic function was demonstrated [3,20]. Two important meta-analyses were conducted on these trials. Lamba et al. reported a 3–23% increase in LVEF with RFCA in different studies, although significant heterogeneity was found with regard to this outcome [21]. Another meta-analysis of 15 studies investigating the role of catheter ablation in patients with frequent PVCs, with a total patient number of 712, demonstrated an approximately 8% recovery in LVEF values in patients with and without PVC-CMP, while the extent of recovery was more than 12% among patients with PVC-CMP at baseline [3]. Left ventricle ejection fraction increased by approximately 3% in our patient group in short-term follow-up. This finding is compatible with findings from the literature.

Premature ventricular complex-induced cardiomyopathy is well known to exert its unfavorable effects on myocardium by restricting the diastole phase of the cardiac cycle in the first place. Preclinical studies demonstrated that tachyarrhythmia causes relaxation abnormalities even when contractile functions are preserved [22]. Evidence suggests that deterioration in left atrial functions and diastolic functions are expected to precede the deterioration in LV systolic functions in tachycardia-induced CMP, being the only manifestation, along with left atrial geometry abnormalities in some cases [23].

In our study, unlike LV systolic functions, which improved only marginally, LV diastolic functions improved significantly during short term follow-up, so that there was no patient with LVDD at follow-up echocardiography. This is notable, because this finding is in agreement with the current knowledge that LV diastolic function is affected earlier, compared with the systolic function, particularly because of inappropriate and inadequate myocardial cellular calcium handling. Right ventricular outflow tract VTs result in a myocardial electrical activation that does not comply with the infrastructure of the conduction system, disturbing interventricular, intraventricular, and atrioventricular synchrony. Shorter cycle lengths associated with tachycardia cause truncation of the diastole at the cellular level with resultant intracellular calcium accumulation, ATP depletion, and ischemia with unfavorable short- and long-term functional and structural effects [24].

It is suggested from studies of RV apical pacing that left atrial remodeling and decreased atrial pump function might be present even in the absence of LVSD [25]. The intermittent and relapsing nature of the tachycardia might result in remodeling and apoptosis at the cellular level. Interestingly, all the patients with LVDD in basal echocardiographic examination also had LVSD in our study. Likewise, these patients who demonstrated recovery of LV diastolic functions also demonstrated recovery of LV systolic functions. These findings might suggest that LVDD may be a marker for earlier stages of myocardial involvement in OT-VTs and indicate reversibility potential of the LVSD. Absence of LVDD, vice versa, might as well indicate irreversibility of tachycardia-induced myocardial involvement, although these hypotheses need to be tested further.

The conventional Doppler parameters of LVDD (E, A, e’) and deceleration time of E-wave displayed significant changes, indicating improvement of LV diastolic functions. The E/e’ ratio decreased significantly in patients with depressed LVEF in contrast to patients with normal LVEF. This finding indicates that LV diastolic functions improve more in patients with compromised baseline LV systolic functions.

Atrial mechanics are closely coupled with ventricular systolic and diastolic functions. The atrial cycle consists of mainly four phases: atrial reservoir, conduit, diastasis, and booster pump phases. The relaxation phase coincides with LV systole, while the remaining phases take place during LV diastole. As an indirect estimate of LV diastolic function and LA cycle, LAAP and LA diastolic volumes might be examined. Left atrial anteroposterior diameters and LAVI indirectly indicate LVDD in the absence of accompanying structural heart disease. Increased LAVI (i.e., above 34 mL/m^2^) is a major criterion for the diagnosis of LVDD [26] Atrial conduit function denotes early stages of LVDD with suction of blood through the mitral valve during LV relaxation. Our patients displayed significant increases in indexed LACV-BSA [11]. This might be the result of restoration of myocardial cellular calcium handling following RF ablation and attenuation of the PVC burden. Booster pump function determines the contribution of LA contraction to LV filling. Booster pump function in our patients decreased significantly at three-months follow-up. This might be the result of improvement in LV suction, while the filling pressures were normal. While the contribution of passive filling increased, the contribution of active filling consequently decreased. Parallel to these changes, LAEF also demonstrated only marginal improvement. Similar improvement in LA mechanics was observed in the subgroup analysis of the patients with PVC-CMP. Left atrium conduit function, however, did not improve significantly. This might be due to decreased LV compliance in patients with PVC-CMP.

To our knowledge, the present study is the first to demonstrate dynamic changes in left atrial volumes from a mechanistic point of view in response to RFCA in patients with PVCs originating from OT. It is remarkable that RFCA substantially improved LA mechanics even in short-term follow-up after RF ablation. This is indicative of an “PVC-induced-atriomyopathy” which might be an earlier manifestation of cardiac involvement in patients with high PVC burden and an early marker of PVC-CMP with LVSD. Frequent PVCs were found associated with CMP even when the average heart rates of the patients were similar to those of healthy controls. Dyssynchrony is proposed as a more probable mechanism than a problem in calcium handling in PVC-CMP. PVC-induced atriomyopathy might be an important pathway leading to reverse remodeling and consequent LVSD in these patients [1]. The current recommendation is to preserve catheter ablation for patients with a PVC-burden level expected to cause LVSD according to the literature, intractable symptoms, or LVSD resistant to drug therapy. This study suggests PVC-atriomyopathy as an early marker of myocardial involvement. Surveillance for PVC-atriomyopathy in patients who are not yet planned to undergo RFCA might support earlier interventions in this patient population.

The findings from our study should be confirmed in larger sets of patients with higher arrhythmia burden and overt LVSD, and longer follow-ups are required to draw more definite and convincing results.

### Limitations

Our study has some limitations. First of all, this study had a small sample size and arose from a single center. Second, we did not perform magnetic resonance imaging (MRI) or 3D echocardiography to assess left ventricle ejection fraction and left atrium volumetric measurements. However, two-dimensional echocardiography is an accepted method for the evaluation of LA and LV functions and is highly correlated to MRI and 3D echocardiography. Another limitation of our study is that patients included in the study included patients with and without normal left ventricular function. Long-term follow-up is indeed important, and we plan to examine long-term changes in atrial functions in the long run too. However, we think that the fact that the favorable changes in LA dynamics were observed even in short-term follow-up is a very significant finding and is worth being reported separately. Lastly, besides premature ventricular contractions, no other ECG variables were investigated related to cardiovascular mortality. A prospective designed study examining the relationship between the ECG variables and cardiovascular mortality should be the aim of a study in the future.

## 5. Conclusions

Disturbance in left atrial mechanics assessed by left atrial volume measurements might be an early manifestation of PVC-CMP as well as a marker of clinical benefit from RFCA, even when LV systolic functions are preserved.

## Figures and Tables

**Table 1:** Volumetric indices of left atrium function (Modified from Hoit et al.).

Measure	Measurement	Interpretation
Left atrium volume index (Maximum left atrium (LA) volume indexed to body surface area (BSA))	Left ventricle end-systole, before mitral valve opening	Global estimate of LA functions and indirect measure of left ventricular diastolic functions (LVDD) and filling pressures
Left atrium conduit volume indexed to BSA	Left ventricle (LV) stroke volume—([left atrium volume index (LAVI)−LAVmin])/BSA)	Estimate of passive phase of diastolic functions and indirect estimate of left ventricle relaxation and suction effect (corresponds to E wave of mitral inflow sample)
Left atrium booster pump volume indexed to BSA	LAVI—LAVmin/BSA—LACV	Estimate of active phase of diastolic functions, contribution of atrial systole to LA emptying and indirect measure of left ventricle late filling (corresponds to A wave)
Left atrium ejection fraction (LAEF)	(LAVI—LAVmin/BSA)/LAVI	Global estimate of LA functions and LA reservoir function
Left atrium passive ejection fraction	LACV/LAVI	Estimate of LA conduit function
Left atrium active ejection fraction	Left atrium booster pump volume (LABPV)/LAVI	Estimate of LA booster pump function and indirect measure of compromise in left ventricle passive filling

**Table 2:** Basal characteristics of the study population.

Age	43 ± 3
Gender (Male/Total)	32/61 (52.5%)
Weight	71 (50–98)
Height	167.4 ± 7.2
Syncope	0/61
Maximum QRS duration	140.5 ± 9.5
Left ventricular Ejection Fraction	53 ± 7
Ventricular premature complex burden in 24-h Holter Monitor	20 (9–33) %
Calcium channel blocker use	27 (44.3%)
Beta blocker use	33 (54.1%)
Amiodarone use	13 (21.3%)
Propafenone use	21 (34.4%)
Any antiarrhythmic *	59 (96.7%)
Anti-arrhythmic medication per patient	1.6 ± 0.7
TSH levels	1.2 (0.5–3.68)
Potassium levels	4.4 (3.8–5.4)
Calcium levels	9.66 ± 0.32
Prior history of ablation	6 (9.8%)

* Calcium channel blocker, beta blocker, amiodarone or propafenone.

**Table 3:** Procedural characteristics of the study patients.

Electroanatomic Mapping System	(*n*, %)
Carto	34 (55.73)
Ensite	27 (44.26)
Temporal measures (median [lowest-highest])	
Procedure time (min)	93.44 (45–214)
Fluoroscopy time (min)	11.80 (3–25)
Radiofrequency energy application time (min)	7.89 (2.50–18.30)
Procedural complications	
Tamponade	1(1.63%)
Cerebrovascular accident	1(1.63%)
Hematoma	3(4.89%)
Site of origin (SOO)	(*n*, %)
Right ventricular outflow tract (RVOT)	29 (47.50)
Cusps	20 (32.70)
Aortic–mitral continuity	3 (4.89)
Epicardial	4 (6.52)
Multiple	2 (1.63)
Pulmonary artery	1 (1.63)
Other	2 (3.27)

**Table 4:** Conventional Doppler echocardiography diastolic function indices before and after catheter ablation in patients with and without PVC-CMP.

	Overall Study Group (*n* = 61)			PVC-CMP (*n* = 18)			Normal LVEF (*n* = 43)			*p*-Value *
	before Ablation	after Ablation	*p*-Value	before Ablation	after Ablation	*p*-Value	before Ablation	after Ablation	*p*-Value	
Mitral E velocity (cm/sec)	86 ±15	96 ± 14	<0.05	85 ± 17	93 ± 12	0.031	85 ± 15	97 ± 15	0.000	0.515
Mitral A velocity (cm/sec)	79 ± 12	77 ± 12	<0.05	84 ± 10	78 ± 13	0.029	76 ± 12	78 ± 12	0.000	0.110
E/A ratio	1.1 ± 0.3	2.8 ± 12.8	0.298	1 ± 0.23	6.7 ± 0.23	0.315	1.15 ± 0.25	1.2 ± 0.17	0.344	0.137
DT (msec)	197 ± 24	184 ± 13	<0.05	203 ± 27	192 ± 15	<0.05	194 ± 22	181 ± 10	<0.05	0.812
Ea average (cm/sec)	10.2 ± 2.8	11.4 ± 0.2	<0.05	8.8 ± 2.2	10.2 ± 2.2	<0.05	10.7 ± 2.9	11.8 ± 2.1	<0.05	0.003
E/Ea ratio	9.3 ± 2.7	8.6 ± 1.7	0.055	11.4 ± 3.3	9.2 ± 1.7	<0.05	8.3 ± 1.8	8.3 ± 1.6	0.877	<0.05
No LVDD	56	61	<0.05	5	18	<0.05	43	43	1.000	<0.05
Grade 1 LVDD	5	0		13	0		0	0		
Grade 2 LVDD	0	0		0	0		0	0		
Grade 3 LVDD	0	0		0	0		0	0		

* *p* values of the difference in change of the parameter in patients with and without PVC-CMP, respectively. PVC-CMP: Premature ventricular complex-induced cardiomyopathy, LVEF: left ventricle ejection fraction, LVDD: left ventricle diastolic dysfunction, DT: deceleration time.

**Table 5:** Changes in systolic, diastolic, and left atrial functions following radiofrequency catheter ablation (RFCA).

	Pre-Ablation	Post-Ablation	*p*-Value
Left ventricular ejection fraction	53 ± 7	57 ± 6	<0.05
Left atrium end-systolic antero-posterior diameter (mm)	38.3 ±3.9	34.8 ± 3	<0.05
Left atrium volume index (mL/m^2^)	18.5 ± 2.8	17.1 ± 2.6	<0.05
Left atrium conduit volume indexed to BSA (mL/m^2^)	18.7 ± 3.7	19.7 ± 3.7	<0.05
Left atrium booster pump volume indexed to BSA (mL/m^2^)	5.5 ± 1.2	4.4 ±1.1	<0.05
Left atrium emptying fraction	0.62 ± 0.04	0.65 ± 0.04	<0.05
Left atrium passive emptying fraction	0.32 ± 0.04	0.41 ± 0.04	<0.05

* *p* value of the constructed General Linear Model for repeated measures.

**Table 6:** Changes in systolic and left atrial mechanic functions with ablation in the PVC-CMP subgroup.

	Pre-Ablation	Post-Ablation	*p*-Value
Left ventricular ejection fraction	43.7 ± 1.6	51.8 ± 1.5	<0.05
Left atrium end-systolic antero-posterior diameter (mm)	39.7 ± 1	36.2 ± 0.7	<0.05
Left atrium volume index (mL/m^2^)	19.8 ± 0.7	18.1 ± 0.7	<0.05
Left atrium conduit volume indexed to BSA (mL/m^2^)	17.1 ± 0.8	18.1 ± 0.9	0.104
Left atrium booster pump volume indexed to BSA (mL/m^2^)	5.9 ± 3	4.4 ± 3	<0.05
Left atrium emptying fraction	0.62 ± 0.01	0.65 ± 0.01	<0.05
Left atrium passive emptying fraction	0.32 ± 0.01	0.41 ± 0.01	<0.05

* *p* value of the constructed General Linear Model for repeated measures.
